# Adhesion response of filopodia to an AFM lateral detachment force and functional changes after centrifugation of cells grown on nanoporous titanium

**DOI:** 10.1016/j.mtbio.2022.100250

**Published:** 2022-04-04

**Authors:** Dainelys Guadarrama Bello, Patricia Moraille, Serine Boughari, Antonella Badia, Antonio Nanci

**Affiliations:** a Laboratory for the Study of Calcified Tissues and Biomaterials, Department of Stomatology, Faculty of Dental Medicine, Université de Montréal, Montréal, Québec H3C3J7, Canada; b Department of Chemistry, Faculty of Arts and Sciences, Université de Montréal, C.P 6128 Succursale Centre-Ville, Montréal, Québec H3C3J7, Canada; c Department of Biochemistry and Molecular Medicine, Faculty of Medicine, Université de Montréal, Montréal, Québec H3C3J7, Canada

**Keywords:** Titanium, Nanotopography, Filopodia, Mechanotransduction, AFM, Centrifugation

## Abstract

Cells sense and respond to mechanical cues from the surrounding substrate through filopodia. Regulation of cellular biomechanics operates at the nanoscale. Therefore, a better understanding of the relationship between filopodia and nanoscale surface features is highly relevant for the rational design of implant surfaces. The objective of this work was to determine the biomechanical contribution of filopodia and their nanoprotrusions to the adhesive interaction of cells with nanostructured surfaces. We have also analyzed the functional changes of entire cells subjected to an external force. MC3T3-E1 osteogenic cells were cultured on polished (Ti-Control) and nanotextured titanium discs (Ti-Nano). An AFM approach was used to measure the lateral detachment force of filopodia. Filopodia on Ti-Nano exhibited higher resistance to a lateral detachment force, which indicates that they adhere to the surface with more strength. SEM analysis revealed a restructuration of the cell membrane in response to centrifugation, being more evident on Ti-Nano. Fluorescence labeling also highlighted a difference in the mitochondrial footprint, a cellular compartment that provides energy for cellular processes. Together, these results show for the first time that surface topography can change the adhesive interaction of a subcellular structure that is fundamental in sensing physico-chemical surfaces features.

## Graphical abstract

**Figure:**
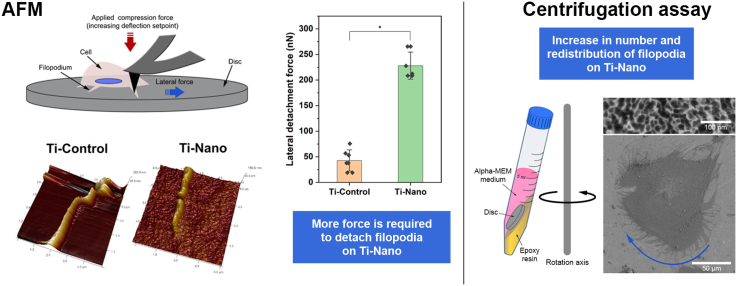

## Credit author statement

Dainelys Guadarrama Bello: Conceptualization, Methodology, Investigation, Writing – original draft preparation, Visualization. Patricia Moraille: Supervision, Writing- Reviewing and Editing. Serine Boughari: Investigation, Writing- Reviewing. Antonella Badia: Supervision, Writing- Reviewing and Editing. Antonio Nanci: Conceptualization, Methodology, Validation, Supervision, Writing- Reviewing and Editing, Funding acquisition.

## 1 Introduction

The different mechanical forces that the human body experiences promote tissue growth and remodeling [1]. These forces are ultimately translated by cells that interact mechanically with their local environment and represent the basic structural and functional unit of the organism [2,3]. Although the structural and molecular aspects of cells are known, their response to mechanical loads and how they convert mechanical signals into biological responses are still not completely defined [4,5].

Cells sense and respond to mechanical cues from the surrounding biological or artificial substrates through lamellipodia and filopodia [6], both actin-rich plasma-membrane protrusions found at the leading edge of cells [7]. During cell migration, filopodia can exert forces on the substrate and act as precursor of focal adhesions (FAs) [8]. This structure is present in almost every moving cell type and its function goes far beyond just probing the surrounding environment. Filopodia define the position of cellular adhesion sites, actin bundles, cell force generation and the formation of new filopodia [9]. The rate of actin filament assembly, and cross-linking will regulate their initiation and elongation [7,10]. Distinct steps have been described in their formation which includes, among others, force dependent adhesion, traction, and retraction. A fundamental aspect of this cell behavior is adhesion, an activity that involves integrin receptor-ligand binding and clustering to form FA complexes [11]. These mechanically link the actin-rich cytoskeleton of cells with the extracellular matrix (ECM) [12]. The cytoskeletal networks of actin, intermediate filaments, and other proteins associated with them determine in large part the mechanical stiffness of cells [13]. Integrins, a family of membrane proteins, act as receptors for cell adhesion molecules via the tripeptide Arg-Gly-Asp (RGD) motif to mediate mechanotransduction to the cytoskeleton [14]. This process transduces mechanical signals from the microenvironment into biological responses [[14], [15], [16]]. The fundamental cell signaling pathways activated regulate diverse cellular activities such as polarization, migration, proliferation, and differentiation [6,17]. These events are influenced by the strength of cell adhesion and are essential for understanding the functioning of cells in the body. They are also critical for the rational design of biomaterials, especially those that are continuously exposed to forces, such as implant loading [18] and blood flow [14,19].

The regulation of cellular biomechanics operates at the nanoscale since cells interact with ECMs comprise of nanoscale constituents such as hydroxyapatite crystals, collagen fibrils, or proteoglycans [20,21]. This also applies to nanostructured medically relevant materials and for this reason, there has been a focus on nanotopography as a tool to improve cell adhesion and activity [14,22]. Therefore, a better understanding of the biomechanical relationship between filopodia and nanoscale surface features is highly relevant for improving implant surfaces.

Real-time force measurements are complex to determine. Different approaches have been used to estimate the cell adhesion strength and quantify the mechanical properties of cells [17]. These include the use of techniques like atomic force microscopy (AFM), optical stretching magnetic twisting cytometry, micropipette aspiration and acoustic radiation-induced deformation, as well as shear forces to detach the cells (e.g. spinning disks, centrifugation, and flow chambers) [23]. Albuschies and Vogel [24], using flexible silicon nanowires (NWs), indirectly estimated the traction force exerted by filopodia from de deflection of the NWs by scanning electron microscopy (SEM) after fixation of the cells. The dynamic behavior of the F-actin present in filopodia has also been investigated on traction and retraction force exerted by live cells, using optical trap and simultaneous optical tweezers and confocal laser - scanning measurements [8,25]. AFM is a unique, high-resolution tool that has been extensively used to study cellular adhesion forces from the single-molecule level to the entire cell [26]. An approach commonly reported in literature is a cantilever tip with an immobilized cell as a measuring probe [13,26]. This technique, known as AFM-based single-cell force spectroscopy (SCFS), is an ultrasensitive method for quantifying cell adhesion forces of single cells. Cellular adhesion force can be measured from the degree of cantilever deflection during cell retraction [26,27]. A similar strategy involves bringing a protein-coated cantilever onto a cell firmly attached to the substrate and then retracting the cantilever [13]. However, these AFM approaches measure the adhesion force exerted by the entire cell and give no information about the contribution of specific subcellular structures to the force.

In previous studies, we have demonstrated that a nanoporous surface induces the formation of more filopodia with abundant nanoscale lateral protrusions that contour the walls of the nanopores [12]. It has been suggested, but not demonstrated, that these distinctive filopodia together with the formation of larger FAs contribute to the overall adhesion strength of the cell. Filopodia traction and retraction have been studied [8,25], but their adhesion, that represents a major component of the filopodia mechanical sensing function has not. In this study, we have determined the biomechanical contribution of filopodia and nanoprotrusions to the adhesive interaction of cells with the surface. These events occur at the subcellular level and measuring forces at the nanoscale is particularly challenging. In fact, to our knowledge, direct measurement of the adhesion force of filopodia in response of a nanotopography, has never been reported. We have adapted an AFM approach used to measure the lateral detachment force of bacteria [23,28] to compare the adhesion forces exerted by filopodia on polished control and nanostructured titanium surfaces. A polished surface was selected to eliminate any topographical features which could confound the contribution of the nanotopography by creating multilevel topography. In order to evaluate the impact of the surface on the adhesion force of the entire cell, we have also analyzed the structural and functional changes exhibited by cells when subjected to an external centrifugal shearing force that does not inherently cause cell damage. Mitochondria are an essential component of all cells in the body that provide energy to perform biochemical reactions and different cellular processes [29]. Because extracellular mechanical factors and the cytoskeleton have an impact on mitochondrial activity, we have also compared the mitochondrial footprint before and after centrifugation [30]. The measurements carried out on fixed cells demonstrate that filopodia and their associated nanoprotrusions induced by the topography exhibit higher resistance to a lateral detachment force, indicating that they adhere to the surface with more strength. Centrifugation results demonstrate an increase in the number of filopodia associated with membrane changes in response to the nanotopography. We also found that the nanoporous surface plays an essential role in regulating mitochondrial networks. Altogether, our combined approach highlighted the profound impact of the nanoporous surface on the adhesion strength of filopodia, and on mitochondrial functionality.

## 2 Materials and methods

### 2.1 Surface modification

Commercially pure grade II titanium discs (12 ​mm diameter x 2 ​mm thickness) (Firmetal Co., Ltd., Shanghai, China) were first polished in three stages as previously described [12] using silicon carbide abrasive paper followed by a Texmet carpet with MetaDi fluid, and 9 ​mm diamond suspension. Finally, a MicroFloc carpet with distilled water and MasterMet SiO_2_ solution was used. The polished discs were rinsed with distilled water in an ultrasonic bath and subsequently cleaned in 70% ethanol and dried with air. Oxidative chemical treatment was used to generate a nanoscale surface topography (Ti-Nano). The polished discs were immersed in a solution of equal volumes of concentrated H_2_SO_4_ (98% mass fraction) and 30% H_2_O_2_ at room temperature (RT) for 1.5 ​h as detailed elsewhere [12,31]. Polished surfaces were used as controls (Ti-Control).

### 2.2 Surface characterization

The Ti-Control and Ti-Nano surfaces were imaged using an ultrahigh-resolution scanning electron microscope (SEM) Regulus 8220 (Hitachi, Ltd., Tokyo, Japan) operated at 1 ​kV. Images were obtained in deceleration mode with a combination of signal from secondary and backscattered electrons without coating the sample. The working distance was around 1.5–3 ​mm. The average pore diameter was measured using ImageJ (http://imagej.nih.gov/ij/features.html).

### 2.3 Cell culture

The Ti-Control and Ti-Nano discs were sterilized using 70% ethanol and UV light for 2 ​h before seeding the cells. MC3T3-E1 (mouse calvaria-derived osteogenic) cells from American Type Culture Collection (ATCC) were cultured in Alpha Minimum Essential Medium with Earle's salts, l-glutamine, ribonucleosides, and deoxyribonucleosides (α-MEM) supplemented with 10% fetal bovine serum at 37 ​°C in a humidified atmosphere with 5% CO_2_. A cell density of 10,000 was plated on Ti-Control and Ti-Nano discs placed in 12-well plates. The cells were grown for 24 ​h.

### 2.4 AFM imaging

Images of attached cells were obtained by AFM using a Dimension Icon NSV scanning probe microscope (Bruker Nano Surfaces, Santa Barbara, California, USA) operated in contact mode in 0.1 ​M sodium phosphate buffer (PB), pH 7.3 ​PB after fixation for 1 ​h at 4 ​°C in 2.5% glutaraldehyde. A silicon nitride tip and cantilever (ScanAsyst Fluid probe, Bruker AFM probes, Camarillo, California, USA) with a low spring and high sensitivity (nominal spring constant ​= ​0.7 ​N/m) was used. The ScanAsyst Fluid is a probe that has a dull tip ideal for force measurements and imaging extremely delicate samples in fluids. The silicon nitride cantilever has a triangular geometry and a back side coating of reflective gold. The tip is connected to the base of the cantilever by an irregular quadrilateral pyramid. The tip radius has a nominal value of 20 ​nm with a maximum of 60 ​nm with a height that varies between 2.5 and 8 ​μm with front, back and side angles from 15 to 25° (According to manufacturer). To select the appropriate filopodia, cells were imaged at a scan size of 40 ​μm^2^ and a scan rate of 1 ​Hz with 128 pixels by line resolution and low set point.

Only those filopodia with a length of at least 5 ​μm, a diameter between 400 and 800 ​nm, and a height of 100 ​nm or more were selected, eliminating width and height as possible variables in the respective force measurements. These dimensions were abundant and readily apparent and therefore we opted to select this. A more refine search using the contact mode was eliminated because the damage it can cause and the time it requires. The tip's scan direction relative to the filopodia was 90° ​± ​5°. Selected filopodia were distanced from other structures (e.g., other filopodia or cell bodies) by a minimum of 5 ​μm to avoid direct contact with other obstacles during measurements. In order to achieve reliable force measurements, all measurements were performed on filopodia displaying similarsize as well as overall orientation.

### 2.5 Lateral detachment force quantification. Force calculation

**Fig. 1:**
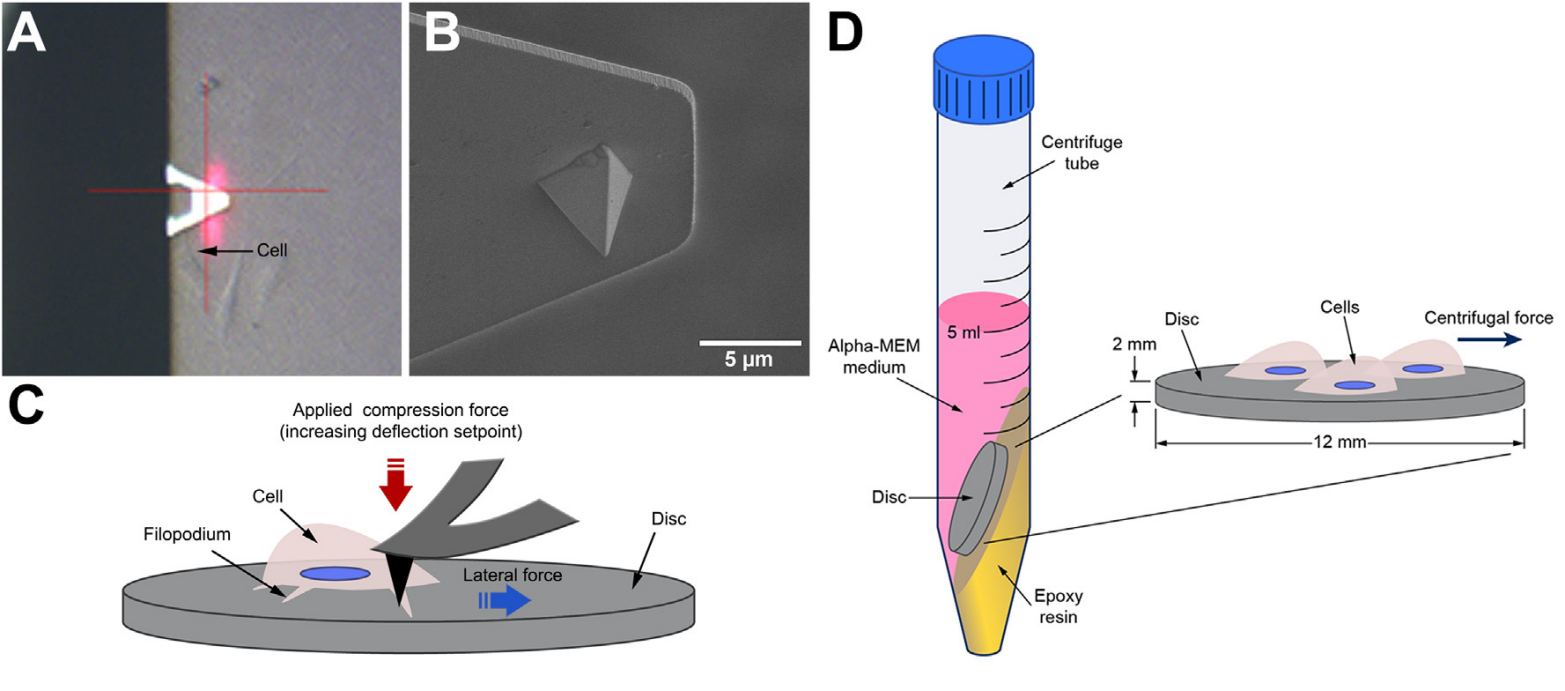
(A) Image from the optical camera showing the top view of the AFM cantilever scanning a cell. (B) SEM micrograph of the lateral view of the pyramidal silicon nitride tip. (C) Schematic representation of the lateral view showing the direction of the compression and lateral forces applied to move the cell. (D) Schematic representation of the disc arrangement for the centrifugation assay.

During the force measurement, the AFM tip (Fig. 1A and B) was scanned across the filopodium at a speed of 1 ​Hz with successive increases of the deflection setpoint with the slow scan axis disabled, therefore ensuring the incremental force was consistently applied along the same scan line until the filopodium detached (Fig. 1C). This force can be calculated using Hooke's Law. However, according to Deupree and Schoenfisch [28], when the probe interacts with a large feature, in this case, a filopodium, the interaction between the tip and the feature will occur on the side of the probe. The equation to describe this interaction and calculate the force is as follows (Eq. 1):

(1) $$F_{lat}=kSV_{total}\text{sin}\left(\theta +\Phi \right)\cos\;\theta$$

where the F_lat_ is the lateral detachment force (nN), k and S are the spring constant (nN/nm) and sensitivity (nm/V) of the applied cantilever, respectively. For each specific AFM probe used, k was determined by performing a thermal tune in air and Lorentzian fitting and S by acquiring a force-distance curve against a clean sapphire substrate. θ and Φ angles are parameters of the probe geometry and cantilever orientation. V_total_ is the total vertical deflection of the laser beam detected by the position-sensitive detector and is directly correlated to the total compression of the cantilever. Three independent experiments (different culture and substrate preparation) were conducted. A total of 9 individual filopodium from 3 different replicas were analyzed for each condition.

### 2.6 Centrifugation assay. Functional changes on cells

We used a modified centrifugation cell adhesion assay that applies controlled force to the adherent cells in the same direction as the force quantification approach described previously. After 24 ​h of culture, cells cultured on Ti-Control and Ti-Nano discs on the 12-well plates were placed directly into 15 ​mL tubes with 5 ​mL of α-MEM medium supplemented with 10% fetal bovine serum at 37 ​°C as represented in Fig. 1D. The discs were placed in a tilt position (15°) to ensure that adherent cells only received a shear centrifugal force. A resin was polymerized at the bottom of each tube with the desired angle, the minimum inclination that was able to keep the disc at the right position through the experiment. Then, the tubes were placed into a centrifuge (Centrifuge Allegra™ X-22R, Ontario, Canada), and cells were centrifuged for 30 ​min at a speed of 1900 g.

### 2.7 Morphology, cell number and FAs

Following centrifugation, cells were fixed for 30 ​min at 4 ​°C using periodate-lysine-paraformaldehyde in PB. Discs without centrifuging were used as control. Then, cells were washed in PB and permeabilized with 0.5% Triton X-100 in PB for 10 ​min. 5% skim milk in PB was used to block nonspecific binding sites for 1 ​h. Cells were incubated for 2 ​h with the specific primary antibody (1:200; Monoclonal Anti-Vinculin Clone hVIN-1 Sigma, MO, USA) and Rhodamine-phalloidin (1:150, Life Technologies) diluted in blocking solution. Alexa Fluor 488 (green fluorescence) conjugated goat anti-mouse was used as a secondary antibody (1:500, Life technologies). All steps of the incubations were performed in a humidified environment at RT protected from light. Between each incubation step, the samples were washed three times (5 ​min each) in PB. Glass slides were used to mount the discs face up, and cell nuclei were stained and mounted with mountain medium containing DAPI (Prolong antifade 4′,6-diamidino-2-phenyl-indole, dihydrochloride, Molecular Probes, Invitrogen) covered with round-glass coverslips. The samples were analyzed with a Zeiss Axio Imager M2 Optical Microscope (Carl Zeiss, Jena, Germany). A 63× objective was used to acquire high-magnification immunofluorescence images to study the cytoskeleton distribution, area, and FA formation on the cells. For each substrate, more than 30 individual cells from three different replicates were evaluated. To obtain high-resolution images of the entire disc, images were captured with a large field of view using a 10× objective, and the surface was subdivided into multiple smaller images to capture tiles. Four discs for each condition (16 images of the whole disc) were used to count the total number of cells. In parallel, control and centrifuged cells were fixed for 1 ​h at 4 ​°C in 2.5% glutaraldehyde and subsequently rinsed three times with PB, followed by incubation for 1 ​h in 1% osmium tetroxide at 4 ​°C. Cells were dehydrated through a graded series of ethanol (30%, 50%, 70%, 90%, 95%, and two times 100%) followed by drying in a Leica EM CPD300 Critical Point Dryer (Leica Microsystems Inc., Ontario, Canada). An SEM Regulus 8220 operated at 1 ​kV was used to observe the morphology of cells grown on control and treated surfaces.

### 2.8 Mitochondrial morphology

To label mitochondria, cells cultured on Ti-Control and Ti-Nano for 24 ​h before and after centrifugation were incubated with MitoTracker® probes (200 ​nM) for 45 ​min at 37 ​°C in a humidified atmosphere with 5% CO_2_. Then, cells were washed with α-MEM without FBS followed by fixation for 15 ​min at 37 ​°C using 4% paraformaldehyde in PB. The samples were washed three times (5 ​min each) in PB. Glass slides were used to mount the discs face up, and cell nuclei were stained and mounted with mounting medium containing DAPI (Prolong antifade 4′,6-diamidino-2-phenyl-indole, dihydrochloride, Molecular Probes, Invitrogen) covered with round-glass coverslips. Images were acquired using a 63× objective with a Zeiss Axio Imager M2 Optical Microscope. More than 10 ​cells from three different replicates were analyzed by each condition.

### 2.9 Image analysis

Image J ((http://imagej.nih.gov/ij/features.html)) was used to estimate the number of the FAs, the area, the number of cells, and the mitochondrial morphology. Images were processed as described in Bello et al. [12]. Images from mitochondrial assays were treated following these steps: (as described by A.J. Valente et al. [32]): unsharp mask, CLAHE, and median. Then, images were binarized and skeletonized to analyze the skeleton as described in Fig. 1S (supplemental materials). Mitochondrial network features as branches, footprint, junctions and rods (individuals) were analyzed. All graphs were constructed with Origin Pro 9.2 software (OriginLab Corporation).

### 2.10 Statistical analysis

The Origin Pro 9.2 software was used to determine the statistically significant differences between the means of different groups using a Student's t-test analysis of mean values. Values of p ​< ​0.05 were considered statistically different, while the values above were not different. Data normality was verified using a Shapiro-Wilk test and the Grubbs test was conducted to verify the existence of outliers. All results are presented as the mean value ​± ​standard deviation (SD).

## 3 Results

### 3.1 Characterization of surface topography

**Fig. 2:**
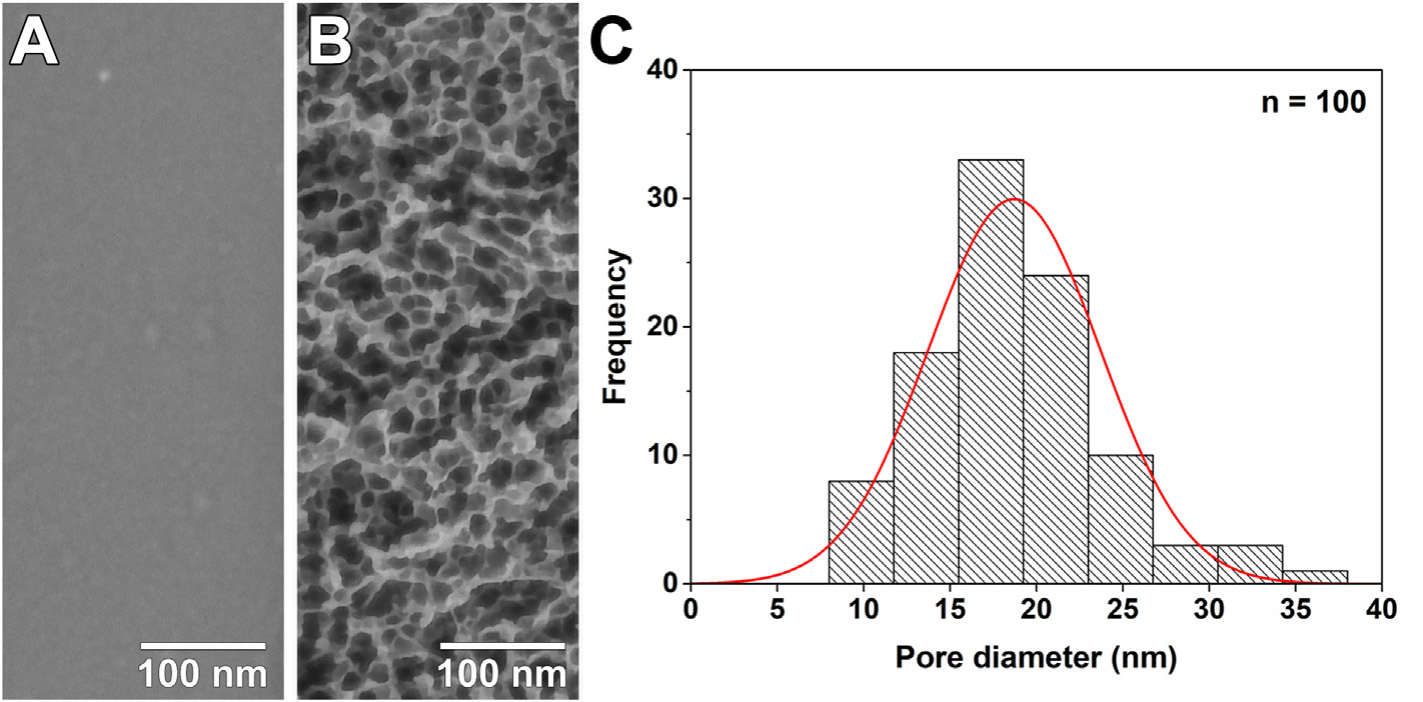
Scanning electron micrographs of the (A) smooth polished Ti surface and (B) nanoporous topography created by the oxidative chemical treatment. (C) Size distribution of the nanopores (*n* ​= ​100).

Fig. 2 illustrates the surface topographies of the polished titanium discs before (Ti-Control) and after (Ti-Nano) oxidative chemical treatment. The Ti-Control showed a smooth surface without topographical features (Fig. 2A). A three-dimensional network of nanopores is observed on the treated surface (Fig. 2B). The mean diameter of the generated nanopores of 19 ​± ​5 ​nm (Fig. 2C) is consistent with previous reports [12,31].

### 3.2 Adhesion force quantification

We measured the cell-substrate adhesion force of MC3T3 cells cultured on Ti-Control and Ti-Nano by contact mode AFM. This method was used to quantify the adhesion strength of filopodia after 24 ​h of culture on the 2 different substrate surfaces.

**Fig. 3:**
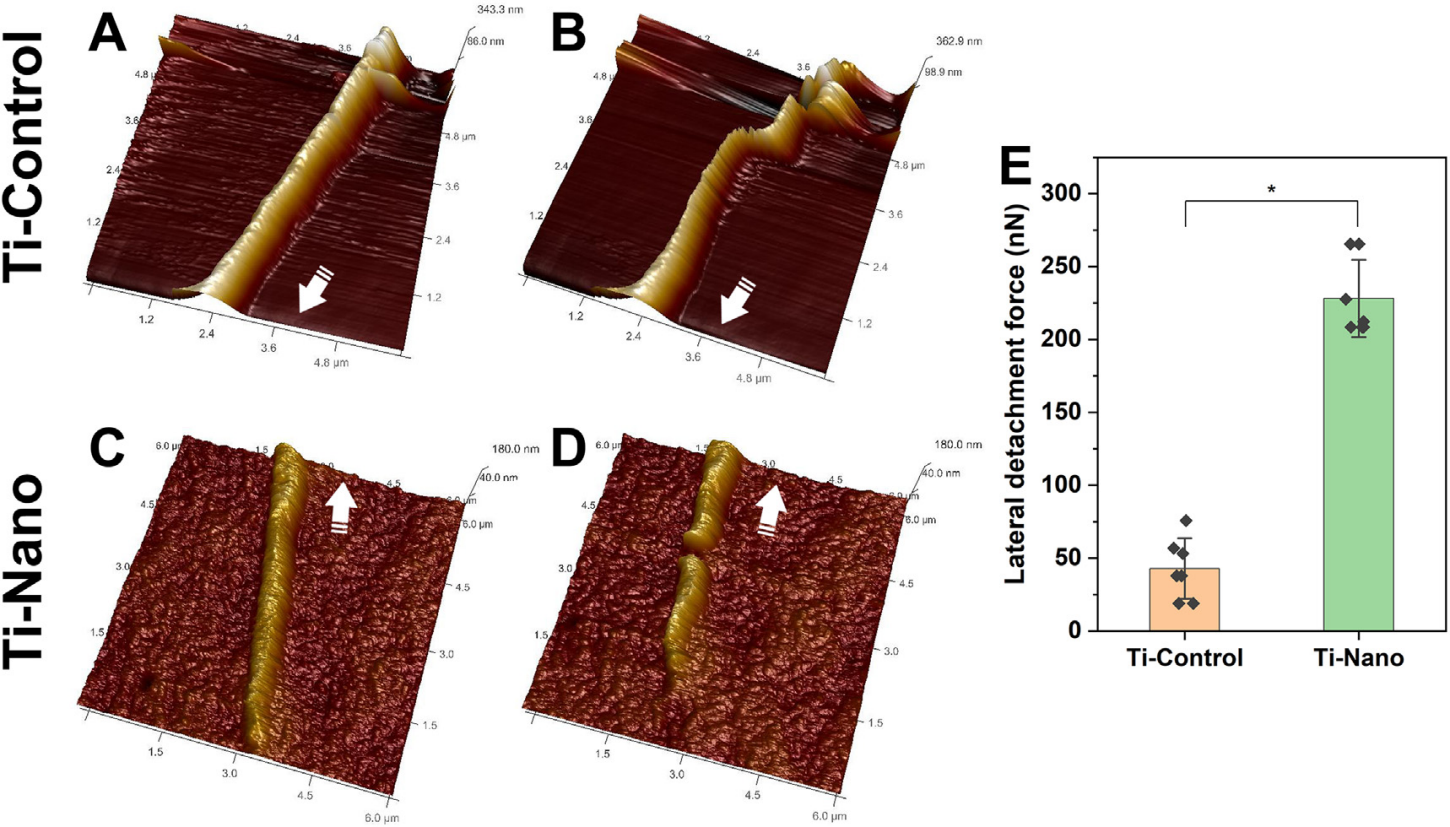
AFM images of filopodia on (A) Ti-Control and (C) Ti-Nano showing the probed regions (A, C) before and (B, D) after increasing the deflection setpoint of the cantilever. In all AFM images arrows represent the direction of the cell body. (E) Quantitative analysis of the lateral force required to detach or break the filopodium on both surfaces obtained after calculation. Dots represent individual data points. Error bars represent the standard deviations, ∗ indicates statistically significant differences (p ​< ​0.05).

The measurements showed that filopodia adhered to the Ti-Nano with more strength than Ti-Control (Fig. 3). Filopodia displaced more readily and using lower cantilever deflection on Ti-Control (Fig. 3A and B), whereas on Ti-Nano (Fig. 3C and D), displacement of the filopodia required significantly higher deflection of the cantilever, in some cases, resulting in tearing of the filopodia. The lateral force increased from 43 ​± ​21 ​nN to 228 ​± ​27 ​nN following oxidative nanopatterning on Ti-Nano (Fig. 3E).

### 3.3 Centrifugation

Centrifugation was employed to investigate the functional changes experienced by cells according to the characteristics of the substrate surface.

#### 3.3.1 Counting and visualizing cells before and after centrifugation

**Fig. 4:**
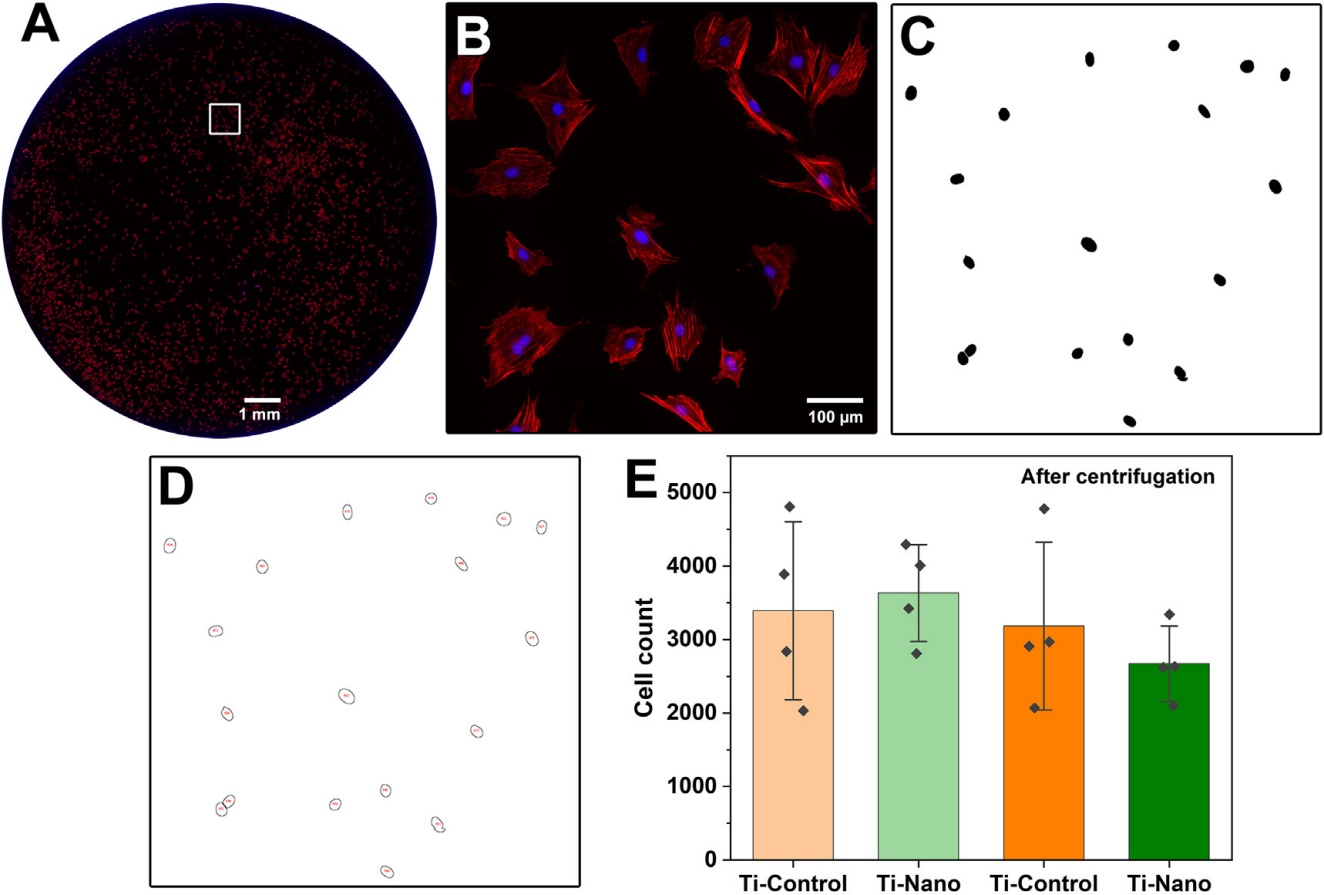
(A) Count from fluorescence microscopy images of cells stained with DAPI (blue) for nuclei and rhodamine/phalloidin (red) for actin. (B) Enlargement of the area outlined by the white square in A. (C) Nuclei maps generated using Image J to automatically calculate (D) the cell number. (E) Number of cells on the polished (Ti-Control) and nanoporous (Ti-Nano) surfaces before and after centrifugation. Dots represent individual data points. Error bars represent the standard deviation. The results show no statistical differences.

The number of adherent cells before and after 30 ​min of centrifugation was measured (Fig. 4). A representative immunofluorescence image used to quantify cells is shown in Fig. 4A and B. Quantitative analysis indicated that centrifugation does not drastically reduce the cell number on Ti-Control and Ti-Nano (Fig. 4E). Most cells remain attached to the surface under the applied centrifugal force.

**Fig. 5:**
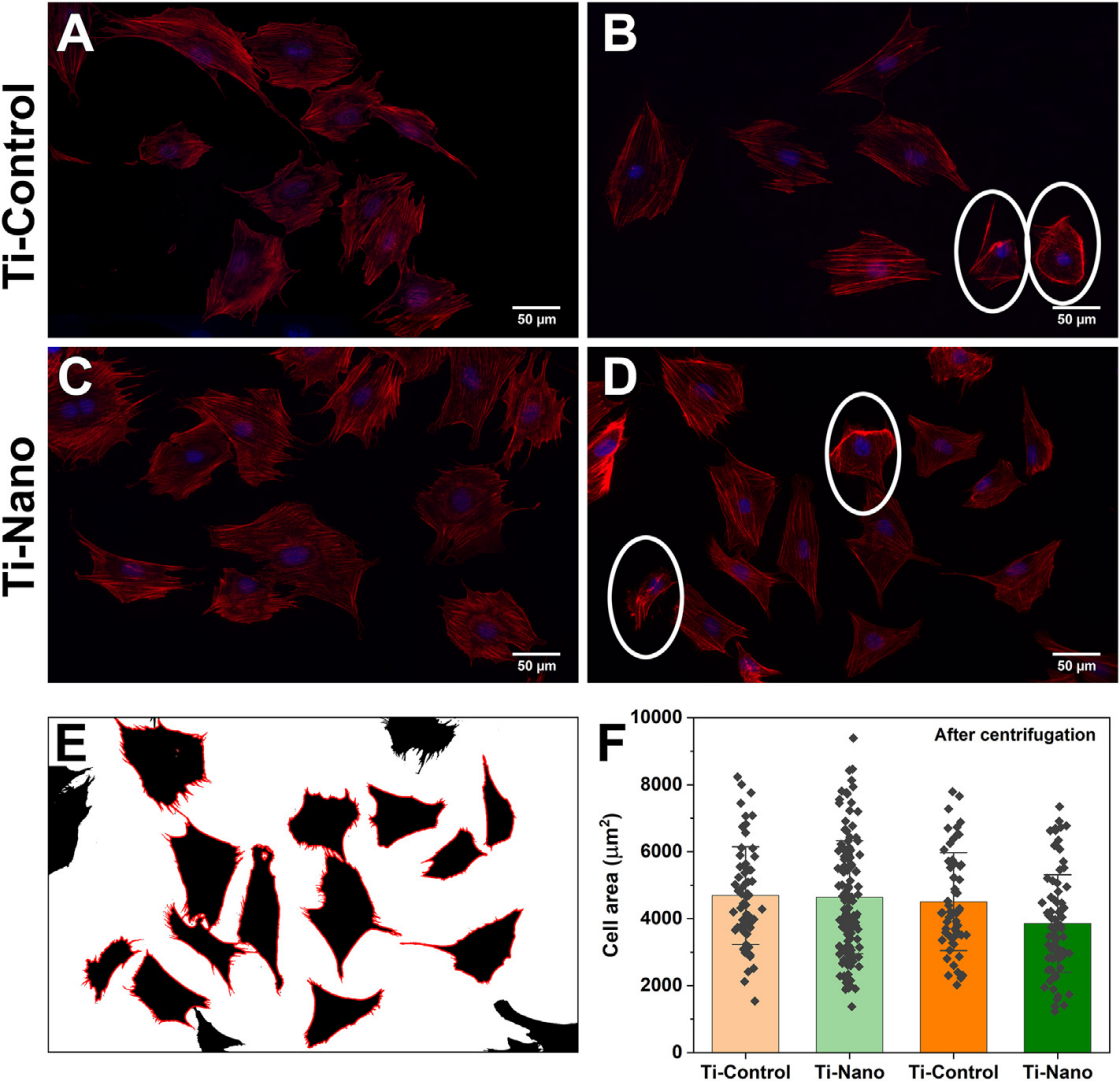
Fluorescence microscopy images of cells stained with DAPI (blue) for nuclei and rhodamine/phalloidin (red) for actin attached on Ti-Control and Ti-Nano (A, C) before and (B, D) after centrifugation. (E) Cells map generated using Image J to automatically calculate the cell area, incomplete cells were excluded from data. Some cells showed regions of peripheral membrane folding (white ovals). (F) The cell areas on Ti-Control and Ti-Nano surfaces before and after centrifugation show no statistical differences under all conditions. Dots represent individual data points. Error bars represent the standard deviations.

To further understand the effect of centrifugation on cells cultured on both surfaces, we analyzed the cell area (Fig. 5). The cell area is not affected by centrifugation (Fig. 5E). We can observe normal variation concerning cell growth. However, some cells showed regions of peripheral membrane folding, suggesting that adhesions or the cytoskeleton were affected by centrifugation (Fig. 5B, D, white ovals).

**Fig. 6:**
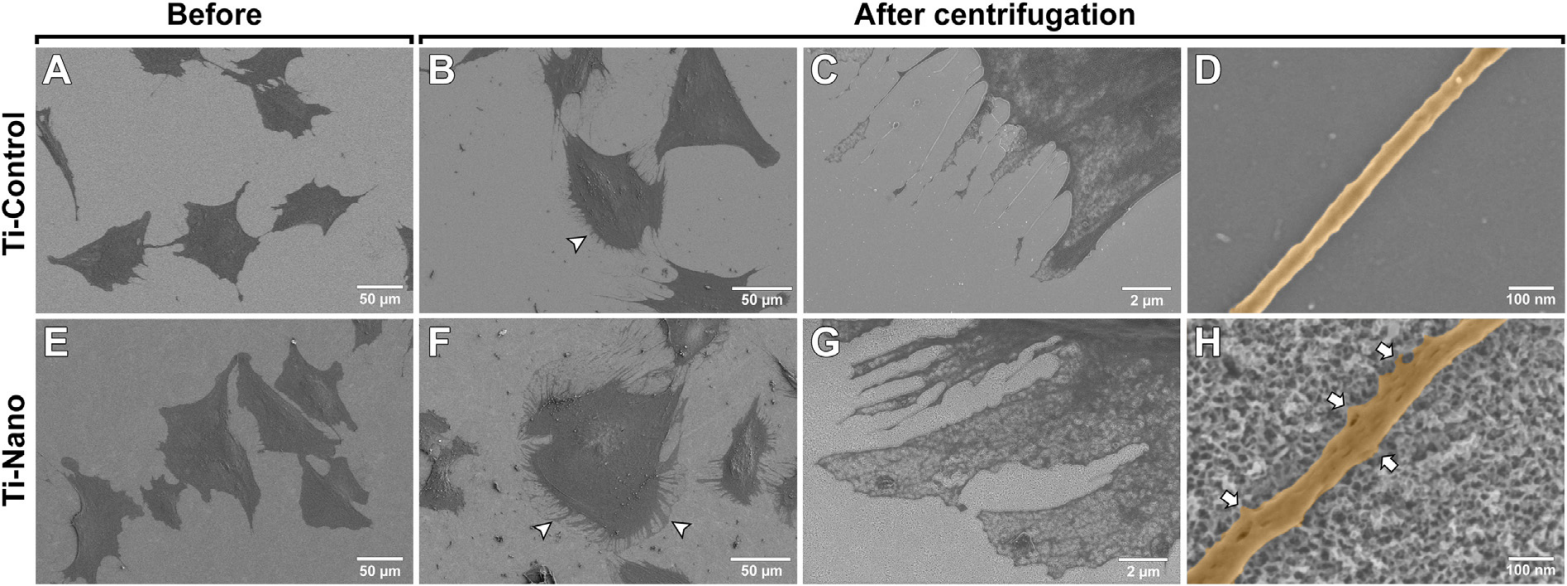
Representative SEM images of cells attached on (A–D) Ti-Control and (E–H) Ti-Nano (A, E) before and (B, C, D, F, G, H) after centrifugation. The distribution of filopodia is represented with arrowheads. High-resolution images of filopodium on (D) Ti-Control and (F) Ti-Nano after centrifugation. (H) Nanoscale protrusions emanating from a filopodium attached to the Ti-Nano surface (arrows).

SEM analysis revealed a restructuration of the cell membrane accompanied by a corresponding change in cell shape after centrifugation (Fig. 6). On some cells, the filopodia concentrated on one aspect of the cells (Fig. 6B, F (arrowheads)), and they were more abundant on the Ti-Nano. On this surface, cells also showed the presence of abundant cell membrane veils, poor in cytoskeleton elements, in response to the centrifugation (Fig. 6G). High magnification images allow us to see that the cell is still developing nanoprotrusions emerging from filopodia in response to the nanotopography (Fig. 6H (arrows)).

#### 3.3.2 Changes in the mitochondrial dynamics in cells after centrifugation

**Fig. 7:**
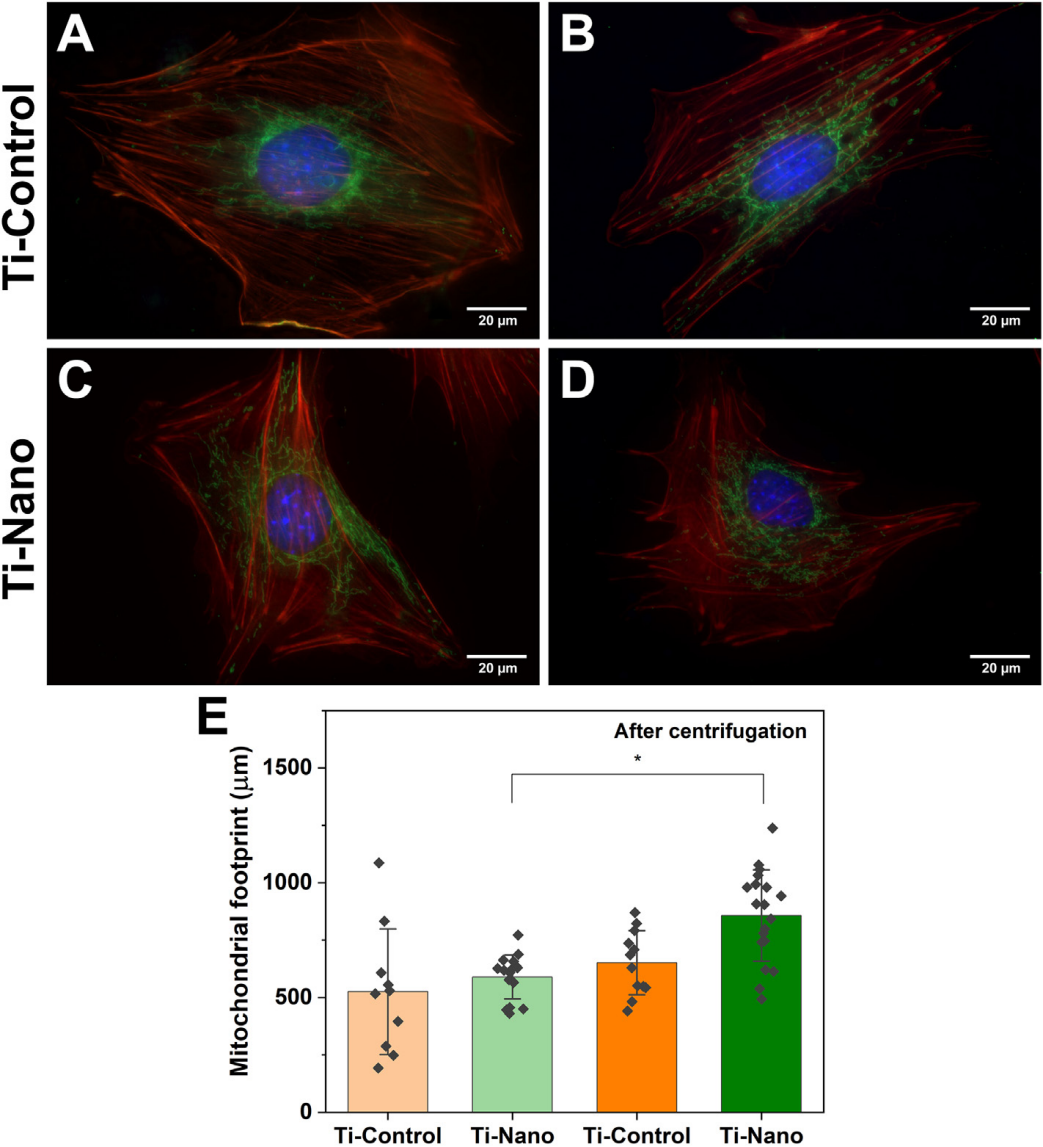
Representative fluorescence micrographs of cells stained with DAPI (blue) for nuclei, rhodamine/phalloidin (red) for actin, and MitoTracker Green (green) for mitochondrial network attached on Ti-Control and Ti-Nano (A, C) before and (B, D) after centrifugation. (E) The surface occupied by the mitochondria. Dots represent individual data points. Error bars represent the standard deviations, ∗ indicates statistically significant differences (p ​< ​0.05).

Surface topography and centrifugation influence the mitochondrial network organization (Fig. 7A–D). The surface of the mitochondria increased. This increase is only statistically significant on Ti-Nano (Fig. 7E). The number of junctions was not affected (Fig. 1S, Supplemental materials).

#### 3.3.3 FAs and cytoskeleton organization

**Fig. 8:**
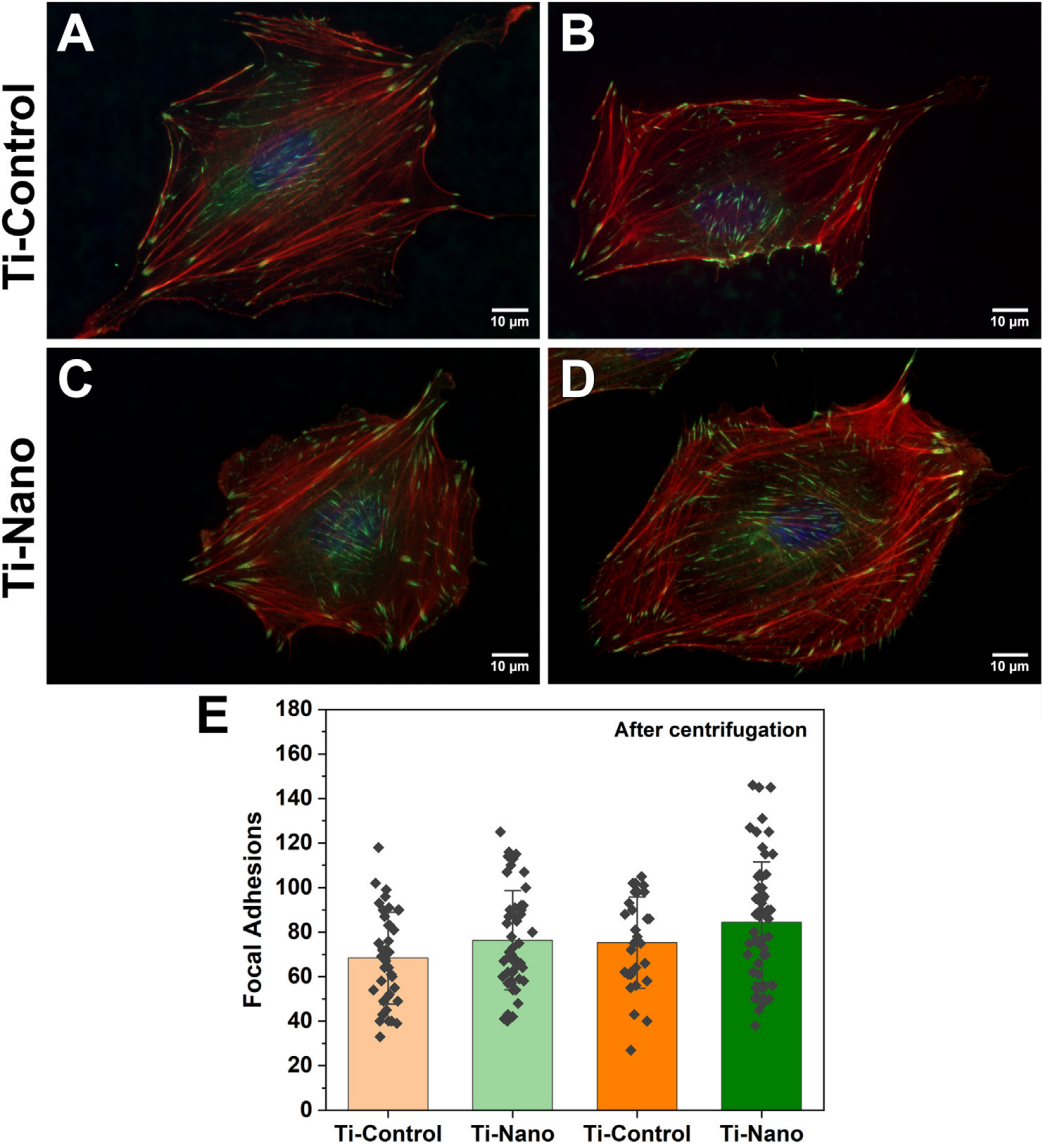
Immunofluorescence images of cells stained with DAPI (blue) for nuclei, rhodamine/phalloidin (red) for actin, and *Anti*-vinculin (green) for FAs attached on Ti-Control and Ti-Nano, (A, C) before and (B, D) after centrifugation. (E) Number of FAs. Dots represent individual data points. Error bars represent the standard deviations. The results show no statistical differences.

To analyze cell adhesion and morphology before (Fig. 8A, C) and after (Fig. 8B, D) centrifugation, cells were stained with Rhodamine-phalloidin to visualize the cytoskeletal organization and anti-vinculin for FA number. There was a tendency for FAs to increase after centrifugation (Fig. 8E), and the images from Ti-Nano suggested a higher concentration of FAs under the region of the nucleus and its immediate surroundings (Fig. 8D).

## 4 Discussion

While various studies have investigated the effects of the substrate surface topography on the adhesion of entire cell [33,34], very few have focused on the role played by filopodia. Here we show that filopodia extended by osteoblastic cells perceive and respond to the underlying nanoporous titanium surface created by oxidative nanopatterning to locally develop stronger biomechanical relationship with the substrate. The fact that five-fold more force is required to displace/break filopodia on the Ti-Nano surface compared to the Ti-Control indicates a significant higher adhesion. Since lateral nanoprotrusions are only found on Ti-Nano, the observed adhesion differential must partly result from their development. Essentially, these very thin membrane extensions allow filopodia to establish more adhesive interactions with the surface. The more frequently observed filopodia breaks on the Ti-Nano, in comparison with the Ti-Control, may reflect an overall differential in stiffness resulting from the more intimate interaction with the surface and/or the distribution of cytoskeletal elements in them that are independent of the cell fixation used. As we have discussed previously [12], the intimate association of filopodia and their nanoprotrusions with the surface suggests that the mechanism may involve changes in integrin conformation and clustering that alter the dynamic organization of signaling proteins in FAs. Using the same experimental conditions, it was shown an increase in the FA area using vinculin staining and the upregulation of the expression of various integrins responsible for the cell-substrate interaction. The higher adhesion force shown here can be correlated with the above molecular findings, demonstrating that filopodia play a critical role in surface topography sensing.

In this work, we complemented the biomechanical AFM analysis with cytochemical and morphological analysis of entire cells following exposure to an external centrifugal shearing force [23]. García et al. [35], used a centrifugation assay to evaluate the influence of multiple biomaterial surface treatments and protein coatings on adhesion of entire cells. They established a correlation between the adhesive properties of cells and different substrate surfaces. Similarly, a centrifugation assay was used to examine the cell adhesion responses to different ligand densities and demonstrated that ligand clustering increased cell adhesion [36]. Neither of these examples analyzed the role of filopodia and their nanoprotrusions. Unlike these above studies, our results revealed no dramatic effect on cell number and area on both the control and nanostructured surfaces. These findings are not surprising because both the duration and the centrifugal force applied were selected to avoid significant cellular damage, and results could change by increasing the time and/or force of centrifugation. However, our study put in evidence that nanoporosity induces pertinent functional changes during as little as 30 ​min of centrifugation. There was, in general, more filopodia which assumed an overall spiraling orientation with respect to the cell surface on the Ti-Nano. One striking particularity is that membrane reorganization resulted in their preferential concentration on one aspect of the cell. This suggests that cells respond to both the strength and orientation of the force applied, which was not the case on Ti-Control. Another distinctive observation is that filopodia on Ti-Nano maintain nanoprotrusions while they are exposed to force. This reaffirms their importance for the adhesive strength of filopodia and may be very relevant for maintaining cell adhesion under more stringent centrifugal conditions.

The highly dynamic mitochondria provide energy to cellular processes [29], and has the ability to fuse and divide and link to the actin network that responds to mechanical forces [30]. Mitochondrial content and their morphology are difficult to quantify due to the degree of branching and their heterogeneity in length [37]. We observed that the mitochondrial footprint of cells on the Ti-Nano surface was more extensive than that of cells on Ti-Control. However, the number of junctions where branches originate does not appear to be affected by the centrifugation on both surfaces (Fig. 1S, Supplemental materials). Consequently, the size of branches and rods must increase, suggesting a concurrent increase in mitochondria fusion that favors activity across the entire tubular network [38]. This suggests that nanotopography would undoubtedly influence mitochondrial function by having an impact on branching.

FAs link the actin-rich cytoskeleton of cells through integrins with the ECM to mediate major cellular events such as mechanosensing and signaling [39,40], but the mechanism by which mechanical stimuli influence FA development remains unclear. As illustrated by the vinculin labeling, there is tendency for increase in the number of FAs after centrifugation that is more prominent on Ti-Nano. They also appear to concentrate in the membrane under and surrounding the nucleus, an intriguing observation that may relate to the distribution of forces along the cell on Ti-Nano. These observations provide a functional confirmation that the physico-chemical changes induced by oxidative nanopatterning influence the behavior of by FAs and their response to external mechanical stress.

Filopodia are essential membrane protrusions that facilitate cellular sensing and interaction with the environment [25]. Our study offers a new approach for the direct measurement of the filopodium adhesion force and represent a significant advance in nanoscale cell mechanobiology. Because filopodia are important mediators of mechanotransduction and they are affected by surface topography [41], it is important to understand how they respond to forces. There is a paucity of studies that measure forces at the subcellular level and the few available use diverse methodologies making difficult to directly compare our results with that of others.

Some studies had dealt with the adhesion force of entire cells [42,43] or with the traction and retraction forces of filopodia [24,25]. However, it has so far not been possible to reliably measure the force with which filopodia adhere to a substrate, essentially for methodological reasons. The differential adhesion force that we have observed between the smooth and nano surfaces reflects different filopodial dynamics on these two surfaces. It also cannot be excluded that this differential adhesion would have an impact on traction and retraction functions of filopodia. In fact, when filopodial adhesion fails, retraction takes place [9]. Therefore, understanding how topography creates different adhesive strengths is important because it can be exploited for the rational design of biomaterial surfaces that will achieve selective adhesive relationship for optimal cellular responses.

There is still no perfect method for measuring the strength of cell adhesion in terms of sensitivity and reproducibility. In fact, all studies have strengths and limitations, and the important consideration is to keep them in mind when interpreting the results. In our study, measuring the force of filopodia after chemical fixation, certainly needs consideration. However, since both surfaces were tested under the same conditions, the difference in lateral detachment force is meaningful. In fact, fixation immobilized in time and space preexistent adhesive interactions of filopodia on both surfaces. Chemical fixation has also been used in the study of Jörg Albuschies & Viola Vogel [24] to immobilize cells during filopodia traction measurements that resulted in very relevant results. Clearly, the next step will be measuring adhesion forces on live cells, but this will present a challenge because filopodia are dynamic structures that could respond and change during the process of lateral force measurement.

## 5 Conclusions

To our knowledge, this is the first *in vitro* study that has directly examined the adhesive interaction between a subcellular structure and a surface. We have applied a quantitative AFM method to compare the adhesion strength of filopodia on smooth and nanoporous titanium surfaces. The formation of filopodia with nanoprotrusions increases and these adhere with more strength on the nanoporous topography. Therefore, these must make an important contribution to the overall adhesion strength of the cell. We have also distinctly analyzed the structural and functional changes of cells when subjected to an external centrifugal force. The observed changes in the filopodia number and distribution, in the overall size of the mitochondrial network footprint, and the distribution of FAs indicate that cells grown on nanoporous titanium also respond differently to an external force. Together, these results show that surface topography can change the adhesive properties of a subcellular component that is fundamental in sensing physico-chemical surfaces features, and this change affects the cell response to an external force. These findings are particularly relevant for prosthetic devices that are subject to external loads, such as orthopedic and dental implants.

## Funding

This research was supported by the Canadian Institute of 10.13039/100005622Health Research (CIHR) and the 10.13039/501100000038Natural Sciences and Engineering Research Council of Canada (NSERC, RGPIN-2016-04764), the 10.13039/100012013Network for Oral and Bone Health Research (RSBO). DGB is the recipient of a scholarship from the 10.13039/501100000156Fonds de Recherche du Québec−Santé (FRQS, 0000273214). AN hold a Canada Research Chair in Calcified Tissues, Biomaterials, and Structural Imaging.

## Data availability

The raw/processed data required to reproduce these findings cannot be shared at this time due to technical or time limitations.

## Declaration of competing interest

The authors declare that they have no known competing financial interests or personal relationships that could have appeared to influence the work reported in this paper.
