# Supplementary material for: Adhesion response of filopodia to an AFM lateral detachment force and functional changes after centrifugation of cells grown on nanoporous titanium
Source: Mater Today Bio. 2022 Apr 4;14:100250. doi: 10.1016/j.mtbio.2022.100250 (PMC9018134; doi:10.1016/j.mtbio.2022.100250)
Supplement: Multimedia component 1 [file mmc1.docx]

**Supplementary Materials**

Adhesion response of filopodia to an AFM lateral detachment force and functional changes after centrifugation of cells grown on nanoporous titanium

*Dainelys Guadarrama Bello^a^, Patricia Moraille^b^, Serine Boughari^a^, Antonella Badia^b^, Antonio Nanci^a,c^*

^a^ Laboratory for the Study of Calcified Tissues and Biomaterials, Department of Stomatology, Faculty of Dental Medicine, Université de Montréal, Montréal, Québec H3C3J7, Canada

^b^ Department of Chemistry, Faculty of Arts and Sciences, Université de Montréal, C.P 6128 succursale Centre-Ville, Montréal, Québec H3C3J7, Canada

^c^ Department of Biochemistry and Molecular Medicine, Faculty of Medicine, Université de Montréal, Montréal, Québec H3C3J7, Canada

**Corresponding Author**

Antonio Nanci

E-mail: [antonio.nanci@umontreal.ca](mailto:antonio.nanci@umontreal.ca)


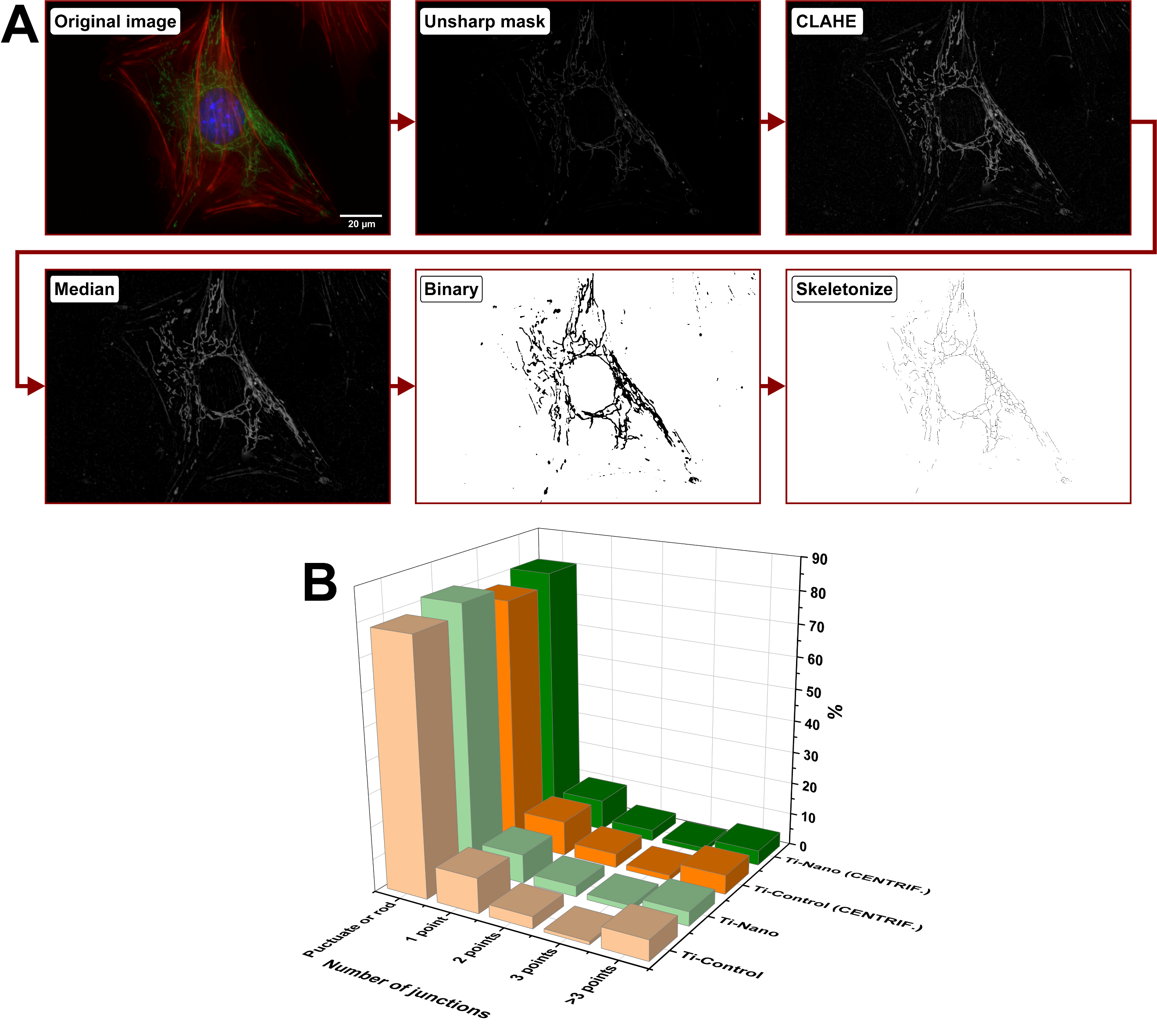


**Figure 1S.** (a) Representative fluorescence micrograph of cells stained with DAPI (blue) for nuclei, rhodamine/phalloidin (red) for actin, and MitoTracker Green (green) for the mitochondrial network followed by the generated images after Image J processing using: unsharp mask, CLAHE, median, binary and skeletonize tools. (b) Number of junctions quantified for each surface before and after the centrifugation. The number of junctions expressed as a percentage was not significantly affected by surface or centrifugation. However, the mitochondrial footprint on Ti-Nano was altered after centrifugation (see Figure 7).
